# Emotional Regulation and Overeating Behaviors in Children and Adolescents: A Systematic Review

**DOI:** 10.3390/bs11010011

**Published:** 2021-01-19

**Authors:** Francesca Favieri, Andrea Marini, Maria Casagrande

**Affiliations:** 1 Department of Psychology, “Sapienza” University of Rome, 00185 Rome, Italy; 2 Department of Dynamic, Clinical and Health Psychology, “Sapienza” University of Rome, 00185 Rome, Italy; andrea.marini.95@outlook.it

**Keywords:** emotional regulation, alexithymia, overweight, overeating obesity, children, adolescents

## Abstract

The worldwide prevalence of obesity has dramatically increased, mostly in children and adolescents. The Emotional Eating theoretical model has proposed that the failure in emotional regulation could represent a risk factor for establishing maladaptive overeating behavior that represents an inadequate response to negative emotions and allows increasing body-weight. This systematic review investigates the relationship between overeating and both emotional regulation and emotional intelligence in childhood and adolescence, considering both cross-sectional and longitudinal studies. Moreover, another goal of the review is evaluating whether emotional regulation and emotional intelligence can cause overeating behaviors. The systematic search was conducted according to the PRISMA-statement in the databases Medline, PsychArtcles, PsychInfo, PubMed, Scopus, and Web of Sciences, and allows 484 records to be extracted. Twenty-six studies were selected according to inclusion (e.g., studies focused on children and adolescents without clinical conditions; groups of participants overweight or with obesity) and exclusion (e.g., studies that adopted qualitative assessment or cognitive-affective tasks to measure emotional variables; reviews, commentary, or brief reports) criteria detailed in the methods. Cross-sectional studies showed a negative association between emotional regulation and overeating behavior that was confirmed by longitudinal studies. These findings highlighted the role of maladaptive emotion regulation on overeating and being overweight. The relationship between these constructs in children and adolescents was consistent. The results indicated the complexity of this association, which would be influenced by many physiological, psychological, and social factors. These findings underline the need for further studies focused on emotion regulation in the development of overeating. They should analyze the mediation role of other variables (e.g., attachment style, peer pressure) and identify interventions to prevent and reduce worldwide overweight prevalence.

## 1. Introduction

Obesity is a heterogeneous syndrome characterized by multifactorial etiology and a substantial imbalance between the assimilated and consumed calories [1]. This clinical condition, commonly defined through body mass index (BMI) [2], is often associated with dysfunctional food-related habits also influenced by individual characteristics [3].

According to the World Health Organization (WHO), the prevalence of obesity in the general population is about 13% [4,5]. The prevalence of obesity in children and adolescents (6–19 years old) has reached around 18% for females and 19% for males [6]. These results have legitimized the definition of the “obesity epidemic” [7] influenced by the maladaptive eating behaviors related to overeating. They represent a risk factor [8] for the increase of body weight and the development of many chronic diseases (e.g., cardiovascular diseases, musculoskeletal complications, diabetes, and cancer; [9,10]).

The excessive intake of foods (i.e., overeating), high in fats and sugars, characterized by an increase in the frequency of intake and quantity of foods consumed, is directly linked to the increase of body weight, causing all the health risks associated with the overweight condition [11]. Several hypotheses have been advanced to explain the onset and development of overeating. Some data reported maladaptive eating behaviors to the current cultural context where the excess of energy-dense foods coexists with low energy-cost availability [12,13,14]. Other authors have hypothesized that overeating is a consequence of cognitive-behavioral conditioning processes [15,16,17], attentional bias for food [18], or altered reward mechanisms that compare it to addiction behaviors [19,20].

The exponential worldwide increase in obesity focused researchers’ attention on the physiological and psychological etiopathogenesis of overeating behaviors [21,22]. Indeed, alongside genetic and environmental factors [23], emotional dysregulation and impairment of interoceptive awareness have been explored as risk factors for overeating and obesity [24,25]. Moreover, in the general population, emotional dysregulation has been associated with high BMI and maladaptive eating behaviors (i.e., overeating; [26,27]). Accordingly, several studies showed significant emotional regulation differences between individuals with excessive body weight and normal weight [28,29,30].

Some empirical data have shown in young adults that an adequate emotional regulation would ensure healthy food choices and a lower probability of losing control of food intake [31]. Conversely, a low level of emotional intelligence and the presence of emotional dysregulation would represent important risk factors for binge eating behaviors [32,33]. Moreover, high levels of alexithymia, found in patients undergoing bariatric surgery, are associated with low weight loss in post-surgical phases [34]. Emotional eating, commonly defined as overeating in response to a negative emotional activation, [35,36] would result from the loss of control in food intake that arises when the attempt to modulate and mentalize unpleasant effects fails [26,37,38].

The relationship between obesity and interoceptive awareness, defined as the ability to identify and differentiate the individual’s perceptions of their internal states [39], was also the subject of scientific analysis. Indeed, some authors showed that low interoceptive awareness appears in young adults, promoting and maintaining excessive food intake [40,41,42,43].

In the face of several data highlighting the strong relationship between emotional dysregulation and overeating in the adult population, some data described the first two decades of life as critical for developing eating habits [9,44,45]. It has been shown that approximately 80% of adolescents with obesity maintain it as adults [44]. In childhood and adolescence, family contexts can significantly impact the relationship between personality and behavior [46]; the parents’ influence could be particularly incisive on the development of maladaptive eating behaviors [47,48,49]. The attachment relationship is an important factor in explaining the intergenerational transmission of eating habits and emotional development during childhood and adolescence. A responsive family context provides children with a greater understanding of the causes, consequences, and the general nature of socioemotional functioning [50]. From late childhood to adolescence (i.e., 12–18 years), the ability to regulate emotions and the likelihood that emotional decisions will be differentiated based on motivation, emotion type, and social-contextual factors increase [51]. Although management of affects would become more and more sophisticated with development, dysregulated emotion processes could lead children and adolescents to learn and use ineffective strategies that place them at risk of being overweight and maintaining obesity in adulthood.

According to some data showing that maladaptive emotional regulation strategies mediate the relationship between impaired parental quality and emotional eating behavior [52,53,54], this systematic review aims to investigate the relationship between overeating and both emotional regulation and emotional intelligence in childhood and adolescence. In particular, the first goal is to verify whether both cross-sectional and longitudinal studies confirm a relationship between maladaptive eating behaviors and one, or more than one, specific domains of emotional regulation and emotional intelligence, such as cognitive reappraisal, emotion suppression, or emotional awareness. The second purpose is to evaluate whether emotional regulation and emotional intelligence can cause overeating behaviors by considering longitudinal studies. The final aim allows assessing the relationship between BMI and both emotional regulation and emotional intelligence by analyzing cross-sectional and longitudinal studies.

## 2. Materials and Methods

### 2.1. Research Strategies and Information Sources

This systematic review was conducted according to the PRISMA Statement [55] and no protocol recording has been provided.

The research was conducted by using Medline, PsychArtcles, PsychInfo, PubMed, Scopus, and Web of Sciences databases using the following keywords: “Alexithymia”, “Emotion Regulation”, “Emotion Dysregulation”, “Emotion Self-Monitoring”, “Emotion Recognition”, “Emotional Intelligence”, “Emotional Empathy”, “Toronto Alexithymia Scale”, “Alexithymia Questionnaire for Children”, “Emotional Regulation Questionnaire”, “Eating Behavior”, “Eating Disordered”, “Emotional eating”, “External Eating”, “Binge Eating”, “Overeating”, “Obesity”, “Overweight”, “Mindless Eating”, “Emotional Hunger”, “Absence of Hunger”, “Eat to Cope”, “Unhealthy Food Intake”, “Eating Habit”, “Child”, “Pre-adolescence”, “Adolescence”, and “School Child”.

The selection of articles was independently made by two researchers (A.M. and F.F.), and the disagreements were solved by a supervisor (M.C.). The software Zotero (AGPL v.3 License; CHNM, Fairfax City, VR, USA) was adopted to facilitate the selection process. An initial deletion of the studies considering the title and abstract content was carried out according to the adopted inclusion and exclusion criteria.

The last search was carried out on 15 December 2019. No time limit was considered, and all studies published up to the date of research were considered.

Table 1 shows the scripts adopted for the systematic search in databases. In Supplement 1, there is a detail of the search strategies adopted in each database and the number of records identified.

The Zotero software was used to remove the duplicates. According to PICOS [56], the information extracted by each study was: authors, year of publication, country, sample information (N, age, sex, type of population), assessment tools, results on the relation between the variables of interest (see Table 2 and Table 3).

### 2.2. Eligibility Criteria

All the studies focused on the relationship between emotional regulation/emotional intelligence and overeating behaviors in children and adolescents and published in international peer-review journals were selected. No cultural or geographical limits to the selection of studies were considered. Therefore, doctorate dissertations that discussed the relationship of interest were also included.

The following inclusion criteria were adopted: (1) studies focused on general populations of children and adolescents (age range: 7–20 years) without clinical conditions; (2) studies on groups of participants presenting overweight or obesity conditions in the absence of organic metabolic causes or eating disorders; (3) studies that adopted standardized self-report instruments for the assessment of emotional variables: (4) studies that analyzed eating behavior, dietary preferences, or weight status to determine the condition related to overeating.

The following exclusion criteria were adopted: (1) studies not written in English; (2) studies focused on samples that did not include children or adolescents; (3) studies that adopted qualitative assessment or cognitive-affective tasks to measure emotional variables; (4) reviews, commentary, or brief reports; (5) studies that included participants presenting medical (e.g., diabetes, metabolic syndrome, hypertension) or psychopathological (e.g., eating disorders, depression, anxiety) conditions; (6) studies that considered the effectiveness of intervention focused on eating behavior or emotional regulation. Although studies evaluating the effectiveness of interventions on eating behavior or emotional regulation might offer some valuable insights, they have been excluded to prevent some confounding variables from hindering a good analysis of interest constructs.

Only studies meeting all inclusion and exclusion criteria were considered eligible for this systematic review.

### 2.3. Quality Assessment

According to the Cochrane Handbook for Systematic Review [83], adapted to the main aims of this study, a quality assessment was carried out.

The dimensions considered for the assessment were: (1) Selection bias (I): the use of standardized tools for the classification of eating behavior or weight status; (2) Selection bias (II): the controlling of confounding variables during the selection of the sample: (3) Detection bias: the use of standardized instruments for the assessment of the emotional dimensions considered; (4) Attrition bias: incomplete outcomes; (5) Reporting bias: selective results discussed; (6) Other sources of biases. These six dimensions were described for each study as characterized by low (“0”), medium (“1”), or high risk of bias (“2”). After calculating the mean score and multiplying it by 100, each study was categorized as at low risk of bias (lower than 75%) or high risk of bias (higher than 75%) (as in [18]). Then, an analysis of the quality of each item of the assessment was reported.

## 3. Results

### 3.1. Studies Selection

**Figure 1:**
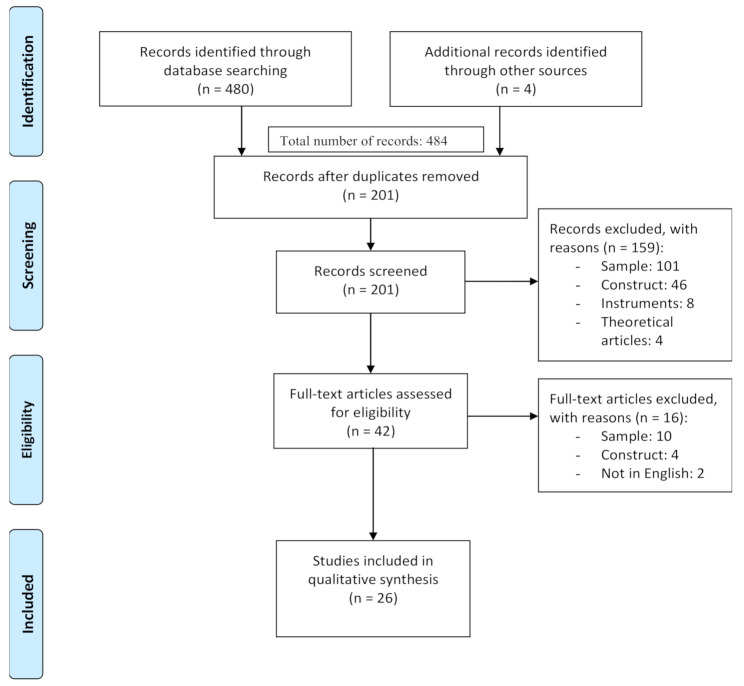
PRISMA Flow Diagram.

The systematic search produced 480 articles. Other 4 articles were identified through other sources such as key journals for the topic or citing papers from key relevant studies. After the exclusion of 279 duplicates, 201 papers were screened. At the end of the selection process, 26 studies were reviewed. Figure 1 shows the process of the selection of the studies.

### 3.2. Qualitative Assessment Results

**Figure 2:**
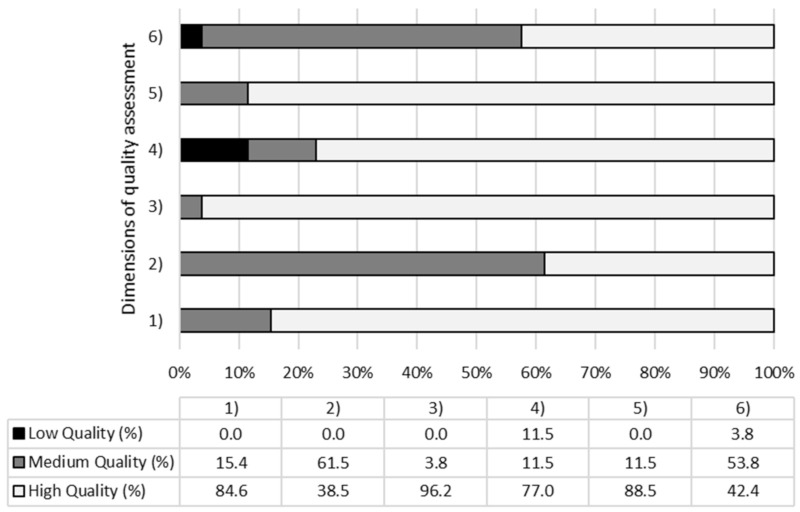
Percentage of studies which presents high/medium/low quality for each dimension assessed. (**1**) Selection bias (I); (**2**) Selection bias (II); (**3**) Detection bias; (**4**) Attrition bias; (**5**) Reporting bias; (**6**) Other sources of biases.

Figure 2 shows the quality assessment results, considering the percentage of the studies obtaining, for each considered dimension, a high, medium, or low quality. Specifically, no study reported a high risk of bias (>75%), a medium-high quality of the studies was found. However, the dimension characterized by the higher risk of bias was the “attrition bias”, typifying studies (11.5%) that reported missing or incomplete outcomes. A lower risk of bias was reported for the items characterizing the “detection bias”, with 25 studies that reported using appropriate instruments to assess emotional variables.

### 3.3. Cross-Sectional Studies

The systematic review identified 20 cross-sectional studies (see Table 2). Sixteen studies were focused on adolescents (age range: 12–20 years), one on children (around 10 years of age), and three studies considered both children and adolescents. Of these lasts, two reported a division considering the mean age, and one indicated only the range of age of the sample (between 11 and 17 years of age) (see Table 2).

Of the 20 studies, one reported a higher percentage of males, seven showed a similar percentage (between 45% and 55%) of males and females; ten studies presented a higher percentage of females; one study considered only females. A study did not report information about the percentage of males and females (see Table 2).

Sixteen studies focused their attention on studying the general population of children or adolescents (see Table 2), and only two studies adopted a classification of participants considering body mass index. Vandewalle et al. [76] analyzed the characteristics of a sample affected by severe obesity, while other authors compared the differences between a group with severe obesity and a group of control with normal weight [59,64,73].

The studies are heterogeneous in the classification of weight status. Wong et al. [77] used the classification suggested by Chen and colleagues [84]; Book and Berant [58] adopted the growth charts of Cole et al. [85] and De Onis et al. [86]; Percinel et al. [73] considered the suggestions of Neyzi and colleagues [87]; the study of Tan et al. [75] adopted the guidelines of the Group of China Obesity Task Force [88]; Gouveia et al. [64] used the WHO Child Growth Standards [89].

#### 3.3.1. Emotional Regulation and Overeating Behavior in Cross-Sectional Studies

A high number of data reported a significant association between emotional regulation and overeating behaviors, regardless of the cultural context where the samples were recruited (see Table 2). However, there is a heterogeneity of the instruments for the assessment of emotional regulation used. The questionnaire most adopted was the Difficulties in Emotion Regulation Scale (DERS), used in five studies [58,64,66,70,73]. Three studies [61,63,76] analyzed emotional regulation with the Questionnaire to Assess Children’s and Adolescents’ Emotion Regulation Strategies [90], and three studies [62,67,69] used the Emotion Regulation Questionnaire (ERQ; [91]). However, other self-report instruments were adopted (see Table 2). The study of Isasi and colleagues [65] used three different scales for analyzing the relationship between overeating and emotional regulation: Soothability [92], Sadness management [93], and Anger management [93] (see Table 2).

For the assessment of eating behavior, the study of Tan et al. [75] adopted BMI as an index of eating behavior; the other studies used self-report measures. Fifteen studies selected a unique self-report instrument [59,60,61,62,63,64,66,67,68,70,71,76,77] one study used two questionnaires [69], and two studies proposed an evaluation with three [74] or four [65] scales.

For the overeating assessment, various instruments were also adopted (see Table 2).

The correlational analysis results (see Table 2) highlighted significant associations between emotional regulation and overeating behaviors [58,61]. Some authors [65] found that higher emotional competencies were associated with higher self-efficacy and adaptive eating habits. Both emotional dysregulation and low emotional intelligence levels were related to a high risk of developing maladaptive eating behaviors [60,64,68,70,71]. Two studies supported a positive correlation between binge eating symptomatology and alexithymia [72], emotional dysregulation, and the absence of emotional awareness [66].

Some authors showed a strong negative association between emotional regulation and emotional eating [76]. Specifically, Lu et al. [69] identified a positive correlation between expressive suppression and emotional eating, and Laghi et al. [67] highlighted an association between emotional expression inhibition and overeating symptoms.

Only Wong et al. [77] reported opposite results than the other studies of this systematic review. Individuals with maladaptive eating behaviors showed higher awareness, expression, and adaptive use of their own emotions. Twelve studies used regression analysis focused on the predictive role of emotional regulation toward eating behaviors (see Table 2). Some studies reported a predictive role of emotional dysregulation [65,66,71], emotional awareness [66], and alexithymia [74] for maladaptive eating behaviors, overeating, and excessive intake of hypercaloric food. Other authors [60,68] identified the negative role of emotional intelligence in predicting the risk of the onset of eating behaviors, bulimic symptoms, and body dissatisfaction.

Five studies [62,67,69,75,76] observed that emotional regulation strategies predict negatively emotional eating [62,76], with the high consumption of hedonic foods [62]. Cognitive reappraisal was the main negative predictor of overeating [67], and expressive suppression was the main positive predictor of excessive and uncontrolled assumption of food in the presence of negative emotions [67,69]. Book and Berant [59] confirmed these results, highlighting a relationship between difficulty identifying and describing feelings and maladaptive eating behaviors associated with the body-weight increase.

Five studies analyzed the differences between groups [61,63,64,73,75]. Two studies compared a clinical group with severe obesity and a group with normal weight [64,73], but only one [73] reported significant differences between groups in emotional regulation. Three studies analyzed the differences between groups with different BMI [61,63,75], showing significant differences between participants with normal weight and overweight or obesity in emotional regulation strategies; furthermore, excessive body weight was associated with overeating behaviors and worst emotional strategies.

#### 3.3.2. Discussion of Cross-Sectional Studies

Despite the heterogeneity of the measures, cross-sectional studies confirmed a relationship between emotional regulation difficulties and overeating behavior. The only results that did not confirm this association [64] may have been influenced by having included only participants overweight or with obesity.

Almost all the studies have focused on adolescence. This period of life has been described as a critical developmental period, characterized by reduced emotional coping [94] and enhanced autonomy from parents’ eating habits. These characteristics could explain some of the observed results.

Various hypotheses have been advanced to explain the relationship between emotional regulation and overeating. Some authors [68,77] have proposed a cultural interpretation bringing back the results to the social idea of “beauty is thinness”. According to this view, overeating would generate social unease and shame for their own body in individuals overweight or with obesity, especially in adolescents. Furthermore, chronic stress would cause emotion regulation difficulties [33,35,95], leading to unhealthy eating behaviors.

Overeating has also been described as an outcome of a maladaptive approach with negative and not-mentalized emotions, particularly anxiety, preceding uncontrolled food intake episodes in individuals that are overweight [61,64]. In the model of Pink et al. [27], it was suggested that alexithymia, which is only indirectly predictive of variation in BMI, would predispose people to higher vulnerability to depressive and anxious symptoms, which would determine impulsive eating behaviors.

Another explanation is proposed by the Escape Theory [96]. This theory suggests that the loss of control in food intake can also be described as the insane attempt to escape from a self-appreciating and ruminative self-awareness [63,96]. Emotional dysregulation, strongly related to depressive symptoms, could inhibit self-efficacy and could facilitate obesogenic behaviors such as sedentary lifestyles [65].

The food-related loss of control could also originate from the absence of mature emotional coping mechanisms, such as distraction, humor, and problem-solving [63]. Several authors [69,71,76] identified a more consistent role of maladaptive emotional coping strategies than functional mechanisms of emotional regulation in determining the overeating behavior. In a study investigating the emotional regulation associated with specific diet patterns [69], a significant relationship was found between emotional eating and emotional suppression, even in individuals in which cognitive reappraisal was not compromised. According to an integrative hypothesis, other results (i.e., [66], in line with an integrative hypothesis, highlighted how both the tendency to adopt emotional suppression and the inability to use cognitive reappraisal could contribute to the development of binge eating symptomatology.

A widely accepted hypothesis suggests the role of attachment style and parents’ characteristics as risk factors for developing maladaptive eating behaviors. A widely accepted hypothesis suggests the role of attachment style and parents’ characteristics as risk factors for developing maladaptive eating behaviors. Some results [59] indicated that the parents’ excessive use of emotional inhibition would reduce the social and affective quality of the relationship. Difficulties in emotional attunement with primary caregivers, especially during the separation-individuation process, could trigger the tendency to resort to maladaptive eating behaviors, such as overeating, to express their emotions [59].

According to this view, distancing [76] and emotional neglect [72] in the parent-child relationship seem associated with affective dysregulation and greater alexithymia. The difficulty in differentiating between the emotional activation and the interoceptive signals of hunger and satiety [97,98] can predispose alexithymic individuals to develop overeating episodes [74]. When maternal control is intrusive, guilty, and rejecting, it could also worsen the children’s need for eating independence [76].

Although the topics of this review have not been addressed in Oceania, South America, and Africa, it is useful to underline that the relationship between maladaptive eating behaviors and emotional functioning seems to persist regardless of the geographical area. Although samples were recruited in different countries with different socio-cultural backgrounds (America, Asia, Europe), all studies showed that the inability to organize one’s emotional world corresponds to a growing risk of being involved in unhealthy eating habits (see Table 2). Assuming that the cultures define what is masculine and feminine, further studies are needed to explore how the gender variable influences the relationship between these constructs. Evidence from both samples in China and Spain agree in suggesting significant differences between males and females, showing how girls presented a higher desire to be thinner, intense preoccupation with weight, body dissatisfaction, and finally more frequent episodes of eating in response to emotional upset [60,69]. Furthermore, it was found in the female group (and not in the male one) an indirect path from suppression to energy-rich food consumption through emotional eating [69]. Compared to the specificity of Western and Eastern contexts, the predictive role of gender on emotional eating could be stronger than that of cultural issues.

### 3.4. Longitudinal Studies

Six studies focused on the relationship between emotional strategies and overeating behaviors adopted a longitudinal design (see Table 3). Five studies were focused on adolescents (age range: 12–20 years) and one on children (age range: 7–9 years).

Three studies included a similar percentage (between 45% and 55%) of females and males; two studies reported a higher percentage of females than males, one study considered only females (see Table 3).

While five studies analyzed the relationship in children or adolescents’ general populations, one study analyzed participants from low-income urban areas.

Only one study [80] reported a BMI classification, using the criteria suggested by Okorodudu et al. [99]. However, these studies did not report an analysis focused on dissimilarities between groups with different BMI.

#### 3.4.1. Emotional Regulation and Overeating Behavior in Longitudinal Studies

Different instruments for assessing emotional regulation were adopted by the longitudinal studies (see Table 3). Moreover, various measures were used to evaluate eating behaviors (see Table 3).

All the studies reported a negative relationship between emotional functioning and overeating, confirming a link between these variables over time.

Five studies [77,78,79,80,82] confirmed a relationship between emotional regulation and overeating behaviors. Specifically, Goldschmidt et al. [77] reported a relationship over time between low emotional awareness and loss of control in eating behavior. Harrist et al. [78] identified a role of maladaptive anger management and emotional inhibition in the onset of emotional and external eating [78], while van Strien et al. [81] highlighted the role of emotional dysregulation in predicting the onset of emotional eating. Orihuela and colleagues [79] focused the attention on the motivational aspects related to eating behaviors. Shriver and colleagues [80] reported a negative correlation between emotional control and emotional eating in adolescents and reported a high risk of overweight occurrence over time in the presence of poor emotional control.

However, some follow-up studies [81,82] did not report a significant correlation between emotional regulation and eating behavior over time.

Regression analysis identified the role of both maladaptive emotional regulation strategies and low emotional awareness in predicting overeating events [77,78]. Finally, Orihuela and colleagues [79] confirmed that emotional regulation influences maladaptive eating behaviors over time. In their study, participants who reported difficulty identifying and expressing emotion had a higher probability of experiencing emotional eating within one year from the first assessment.

#### 3.4.2. Discussion of Longitudinal Studies

Although the presence of mixed results, longitudinal studies confirmed the negative relationship between emotional regulation and overeating and, generally, reported its persistence over time. In longitudinal studies, as in the cross-sectional ones, the main age target was adolescence, described as a critical developmental period in which there is an asymmetry between negative affectivity and available affective regulation strategies [77]. The results add to the growing literature suggesting the role of recurrent use of reactivity and emotional inhibition in the etiology and maintenance of emotional and external eating [78]. Moreover, both the difficulty in understanding and regulating negative emotions were considered causal factors of onset and maintaining dysregulated eating behaviors over time [77].

Vandewalle et al. [81] have analyzed the role of parents in determining emotional eating behaviors. The authors have also reported a predictive role of the mother-child relationship for emotional dysregulation and emotional eating. However, these results were not confirmed by the follow-up assessment. In the transition from childhood to adolescence, there is progressive emancipation from parents [100] and increased social pressure from peers [101]. These aspects allow suggesting a role of social conformism in the prediction of overeating [79].

## 4. General Discussion

Previous reviews have analyzed the association between emotional regulation and overeating behavior [35,102,103,104,105,106]. However, none of these have investigated this relationship in a healthy general population, especially considering childhood and adolescence. Some evidence suggested that compulsive eating behavior and obesity originate from a long asymptomatic history of hypercaloric food consumption [107].

This systematic review analyzed the studies that focused exclusively on non-clinical populations of children and adolescents. Differentiating the analysis of the cross-sectional studies from that of the longitudinal ones allowed reflecting better on the quality and persistence over time of the relationship between emotional competence and maladaptive eating habits during childhood and adolescence, supporting the use of interventions focused on improving one’s understanding and management of negative emotions.

Regarding this review hypothesis, both cross-sectional and longitudinal studies confirmed a correlational and predictive association between reduced emotional competencies and overeating [60,64,66,77]. In particular, distinct domains of emotional regulation and emotional intelligence, such as difficulty describing emotions, lack of emotional awareness, and expressive suppression were described as predictive factors of maladaptive eating behaviors. Finally, significant differences in the emotional regulation ability between groups with different BMI were also found [60,62,74].

Most of the studies provided control of some confounding variables, such as sex, age, and BMI, highlighting the possibility to consider the relationship between emotional abilities and overeating as a complex phenomenon. In fact, this relationship appears to be influenced over time by a plurality of physiological, psychological, and social factors [59,61,77,78,80,81,108]. Future research should clarify the role of many other variables compared to those evaluated in this study.

## 5. Limits

This systematic review presents some limitations. The low number of studies directly focused on the relationship between emotional regulation and overeating restricts the generalizability of these results. Furthermore, the small sample size does not allow generalizing the results of both cross-sectional and longitudinal studies. The higher prevalence of females in the analyzed studies represents another restraint. Some results [59,68] identified significant differences between males and females that should be further investigated. Additionally, the exclusion criteria about the English language and the exclusion of so-called “gray literature” (unpublished works) could have resulted in the exclusion of articles useful in further clarifying the nature of the relationship.

An additional constraint concerns the characteristics of longitudinal studies. Only one study [80] considered an extended four-year follow-up [80]. Other studies have not investigated the persistence of the relationship between these variables over a long time, so it has not been possible to make empirical inferences on how these dimensions change over time. Another limitation is the reliability of emotional regulation measures in children, although studies have generally used validated tests. This limit prompts caution in the interpretation of data concerning children. Lastly, this review has not considered other psychological variables that could modulate the relationship between emotional regulation and excessive body weight. It would be a limitation that makes it difficult to find a univocal interpretation of the results.

## 6. Conclusions

The studies analyzed in the present review has highlighted consistent results on the association between emotional regulation and overeating behaviors in non-clinical populations of children and adolescents. However, some doubts about the temporal persistence of the relationship between effective competence and overeating persist. Further studies are needed to point out the periods of childhood and adolescence that are crucial for the consolidation of maladaptive eating behaviors and to confirm the relationship between emotional dysregulation, overeating, and overweight/obesity during the lifespan.

This review could help further research to achieve new conclusions that can help promote interventions focused on diminishing overeating, overweight, and obesity in the first phases of the development. Early interventions and prevention programs in childhood and adolescence could be important for reducing the ongoing increase in the prevalence of these diseases in the general population. These interventions, characterized by an integrated approach, should consider the emotional world and the individual’s affective regulation abilities, taking into account the different patterns of association that emerge in the development and according to the main characteristics reported by this review. This approach may promote greater effectiveness of the intervention.

Moreover, it would be useful a meta-analysis of the studies that could contribute to a more reliable interpretation of the results.

Although several scientific analyses emphasize the role of other variables, the interdependence between emotional regulation and overeating seems evident. These topics should be further explored through researches that should consider a longitudinal experimental design involving children and adolescents.

## Figures and Tables

**Table 1:** Databases, scripts, and the number of records for each database.

Databases	Scripts	Records
Medline	Since EBSCOHost does not allow the filters TI (Title) and AB (Abstract) to be used simultaneously for the same script, it was necessary to conduct two separate searches. While in the first one, all the mini-scripts were searched with filter TI title; in the second one, the first two mini-scripts were found with filter AB and the third one only with the filter TI. Finally, the two searches were combined through the command “Search with OR”.	101
PsycArticles		6
PsycInfo		132
Pubmed	(((alexithym* [Title/Abstract] OR “emotion* regulation” [Title/Abstract] OR “emotion* disregulation” [Title/Abstract] OR “emotion* self-monitor*” [Title/Abstract] OR “emotion recognition” [Title/Abstract] OR “emotional intelligence” [Title/Abstract] OR “emotional empathy” [Title/Abstract] OR “Toronto alexithymia scale” [Title/Abstract] OR “alexithymia questionnaire for children” [Title/Abstract] OR “emotional regulation questionnaire” [Title/Abstract])) AND ((“eating behav*” [Title/Abstract] OR “emotional* eating” [Title/Abstract] OR “external* eating” [Title/Abstract] OR “binge eating” [Title/Abstract] OR *overeating* [Title/Abstract] OR obesity [Title/Abstract] OR overweight [Title/Abstract] OR “mindless eating” [Title/Abstract] OR “emotional hunger” [Title/Abstract] OR “absence of hunger” [Title/Abstract] OR “eat* to cope” [Title/Abstract] OR “unhealthy food intake” [Title/Abstract] OR “eating habit*” [Title/Abstract])) AND ((child* [Title/Abstract] OR “pre-adolescen*” [Title/Abstract] OR adolescen* [Title/Abstract] OR “school child*” [Title/Abstract]))). The script was completed with the following filters: “Full text”, “Humans”, “English”, “Child: birth-20 years”	65
Scopus	It was used the script in Table 1.	152
Web of Science	Since Web of Sciences does not allow the filter “TITLE-ABS-KEY” to be used simultaneously for the same script, it was necessary to conduct two separate searches. The first two mini-scripts (emotional regulation and eating behaviors) were found with filter TOPIC, and the third one was found with filter TITLE.	226

**Table 2:** Cross-sectional studies investigating the relationship between Overeating Behavior and Emotion Regulation.

Author (Year)	Country	Sample				Instrument 1 (Overeating Behavior)	Instrument 2 (Emotion Regulation)	Relation Between Variables	
		N	Age (Years) M (SD)	Gender (Males)	Typology of Population			Correlation and Regression	Differences between Groups
Bayraktar, 2015 [57]	Turkey	401	Range of age: 18–19	122 (30.4%)	Adolescents	EAT-26	DERS	AERS and EB presented a significant positive correlation	
Book & Berant, 2013 [58]	Israel	90	Patients 16.70 (1.16) / Controls 16.82 (1.30)	0 (0%)	45 HC (BMI < 25) and 45 OWA (BMI ≥ 25) or OA (BMI ≥ 30)	EAT-26	NMR	DDE predicted the ability to *RNEEB* that predicted higher susceptibility to obesity	
Cuesta-Zamora, González-Martíb, & García-Lópeza, 2018 [59]	Spain	382 preadolescents / 380 adolescents	Preadolescents 10.55 (0.60) / Adolescents 13.53 (1.25)	Preadolescents 184 (48.2%) / Adolescents 198 (52%)	Normal population	EDI-3	TEIQue-ASF	TEIQue-ASF presented a significant negative correlation both with EDI-3 and its subscales (BD-EDI-3, DT-EDI-3 and B-EDI-3). EI emerged as a significant predictor of BD-EDI-3, B-EDI-3 and EDI-3-TS both in boys and girls	
Czaja, Rief, & Hilbert, 2009 [60]	Germany	127 (65 LOC+ and 62 LOC-)	LOC+ 10.62 (1.46) / LOC− 10.92 (1.47)	25 LOC+ (41.7%) / 27 LOC− (45%)	Normal population	DEBQK	FEEL-KJ	r FEEL-KJ and DEBQ-K (ExE and EmE) presented a significant positive correlation	LOC+ children reported higher use of mERs for anxiety, anger, and sadness than LOC-
Ferrer, Green, & Oh, 2017 [61]	USA	1556	14.45 (1.62)	778 (50%)	Adolescents	27-item dietary screener	Four-item version of the ERQ	ERQ-ES was a predictor of EmE, lower F&V consumption frequency and greater HF consumption frequency	
Goossens, Van Malderen, Varn Durme, et al., 2016 [62]	Belgium	528	15.08 (1.59)	155 (29.4 %)	Normal adolescents	ChEDE-Q	FEEL-KJ		LOC+ vs. LOC− Regarding mERs, LOC+ adolescents reported more NEG, S-DEV, and RUM, WIT than LOC− ones. Regarding aERs, LOC− used, compared to LOC+, reported more use of P-OA, DIS, PIGH, ACC, CPS
Gouveia, Canavarro, Moreira, 2018 [63]	Portugal	245	14.49 (1.71)	Normal adolescents 62 (51.7%) / Adolescents in nutritional treatment 62 (49.6%)	136 OWA and 109 OA / 125 (51%) patients and 120 (49%) from the community	DEBQ	DERS-SF	DER resulted to be positively correlated with EmE	No significant differences were found for difficulties in emotion regulation, or emotional eating
Isasi, Ostrovsky, Wills, 2013 [64]	USA	602	12.7 (0.8)	253 (42%)	Students	YAQ (Dietary assessment) / YRBS (Physical Activity) / A 6-item scale for S-EHFC / A 6-item scale for S-EBPA	An instrument to assess ER, including: a scale for soothability / A 5-item scale for sadness management / A 4-item scale for anger management)	ER was positively correlated to S-EBPA and S-EHFC that was related to F&V intake ER was a direct predictive factor of snack/junk food intake	
Laghi, Bianchi. Pompili, 2018 [65]	Italy	804	17.45 (1.02)	404 (50.3 %)	Students	BES	DERS (Scale “Lack of emotional awareness” was considered as an independent measure)	ED and LEA resulted to be not only positively correlated to BE, but also its significant predictors. Regarding LEA, it is true only at high levels of NCT	
Laghi, Liga, Pompili, 2019 [66]	Italy	1004	17.9 (0.8)	395 (39.34%)	Students	BES	ERQ	BES-TS and ERQ-ES were positively correlated. ERQ-CR and ERQ-ES were negatively and positively, respectively, predictors of BE	
Li, 2018 [67]	China	784	17.12 (1.32)	382 (48.72%)	Students	EAT-26 (Chinese version)	WLEIS	EAT-26 and WLEIS were positively correlated. EI was a negative predictor of EaDR.	
Lu, Tao, Hou, 2016 [68]	China	4316	Range of age: 11–17	-	Students	FFQ / DEBQ	ERQ	ERQ-CR In boys, it was found a positive correlation with N-DDP. In girls, a positive correlation with N-DDP and a negative one with E-RDP. ERQ-ES In boys, it was found a positive correlation with EmE. In girls, a positive correlation with both EmE and E-RDP and a negative one with N-DDP In girls, E-RDP was predicted by: ERQ-ES → EmE → E-RDP	
McEwen, Flouri, 2008 [69]	England	203	14.04 (1.91)	78 (38.4%)	Students	EAT-26	DERS	DER and EaDS were positively correlated	
Mills, Newman, Cossar et al., 2014 [70]	United Kingdom	222	15.38 (1.05)	123 (55.4%)	Students	EAT-26	21 items-REQ	DER and DEa were positively correlated DER was a predictor of DEa	
Minnich, Gordon, 2017 [71]	USA	1344	18.97 (1.24)	481 (35%)	Students	BES	TAS-20	TAS-20 and BES were positive correlated	
Percinel, Ozbaran, Kose et al., 2016 [72]	Turkey	60	30 patients 14.57 (2.07) / 30 controls 14.73 (1.85)	Patients 4 (13.3%) / Controls 4 (13.3%)	30 patients EO / 30 Controls		DERS		DERS-TS and all its subscale were significantly higher in EO group compared to HC
Shank, Tanofsky-Kraff, Kelly et al., 2019 [73]	USA	200	13.1 (2.8)	92 (46%)	Community sample	EDE interview / Emotional Eating Scale for Children and Adolescents / Eating in the Absence of Hunger Questionnaire	AQC	Alexithymia was a predictive factor of emotional eating, eating-related psychopathology, and eating in the absence of hunger.	
Tan, Xin, Wang, 2017 [74]	China	2042	OWA 15.06 (1.95) / OA 14.50 (1.93) / HC 14.92 (1.80)	OWA 500 (71.43%) / OA 230 (71.65%) / HC 730 (71.50%)	Students OWA: 700 (34.28%) / OA: 321 (15.71%) / HC: 1021 (50%)		CERQ	Greater S-B and RUM were predictors of higher BMI, while greater ACC and “P-REF were predictors of lower BMI.	OA obtained the highest scores on both S-B and RUM scales, and OWA obtained higher scores than HC. HC obtained the highest scores on ACC, P-REF, and P-REA
Vandewalle, Moens, Braet, 2014 [75]	Belgium	110	13.59 (1.64)	47 (42.7%)	Children and adolescents with obesity	DEBQ-child version	FEEL-KJ	mERs and EmE were positively correlated mERs and Adjusted BMI were negatively correlated mERs, but not aERs, was a significant predictor of EmE	
Wong, Ling, Chang, 2014 [76]	Taiwan	1028	16.1 (0.7)	420 (40.86%)	Students divided into 4 groups: UW, NW, OW and OA	EAT-26	Adolescent Emotional Intelligence Scale	EAT-26-TS was positively correlated with EI and its subscales EA, EE and EU	

All the abbreviations can be find in the Abbreviations/Nomenclature section at the end of the manuscript.

**Table 3:** Longitudinal studies investigating the relationship between Overeating Behavior and Emotion Regulation.

Author (Year)	Country	Sample				Instrument 1 (Overeating Behavior)	Instrument 2 (Emotion Regulation)	Relation between Variables	
		N	Age (Years) M (SD)	Gender (Males)	Typology of Population			Correlation and Regression	Differences between Groups
Goldschmidt, Lavender, Hipwell et al., 2016 [77]	USA	588	3 assessment 16.5 (0.4) / 17.3 (0.4) / 18.3 (0.3)	0 (0%)	Normal sample	EAT-26	Two DERS-subscales: EA and ERS	mERs and LEA were positively correlated with LOCef at every year of age. mERs and LEA in adolescents aged 17 and 18 independently predicted LOCef at 18 age	
Harrist, Hubbs-Tait, Topham, 2013 [78]	USA	740	Range of age 7–9 years old	375 (50.7%)	Students	DEBQ	CEMS	Regarding Anger and Worry, each ERea and EI measures were significantly or marginally correlated with ExE and EmE. Regarding Anger and Worry, each measure of ERea and EI were predictive of change in ExE and EmE	
Orihuela, Mrug, Boggiano, 2017 [79]	USA	75	Waves 2 15 (0.97) Waves 3 16 (1.11)	37 (47%)	Students	K-PEMS	ERICA	Wave 3-ER were negatively correlated with CEM, CoEM at Wave 2, and CEM and EEM at Wave 3. Poorer Wave 2-ER predicted CoEM and CEM at Wave 3	
Shriver, Dollars, Lawless et al., 2019 [80]	USA	153	3 assessment 15 / 16 / 19	67 (44%)	Normal sample 68.8% HW / 15.6% OW / 15.6% OB	TFEQ	ERCA	ER at 15-year was positively correlated with DR at 16-year and negatively correlated with EmE at 16-year. Furthermore, DR at 16-year and EmE at 16-year were both positively correlated with BF at 19-year. ER at 15-year was a positive predictor of DR at 16-year and a negative predictor of EmE at 16-year. EmE was a positive predictor of BF at 19 years.	
Vandewalle, Moens, Beyers et al., 2016 [81]	Belgium	81	First assessment 12.86 (1.65) / Follow up 4 months later	36 (44.4%)	Students	DEBQ	FEEL-KJ	Regarding the “Level”, mERs was positively related to EmE, but concerning the “Change”, mERs was not related to EmE.	
van Strien, Beijers, Smeekens et al., 2019 [82]	Netherlands and Finland	105	Wave 1 12.38 (0.28) Wave 2 16.27 (0.30)	52 (50%)	Community sample	DEBQ-C	ERQ / TAS-20	EmE at 12-year was correlated with TAS-20 DIF and tas-20 DDF	

All the abbreviations can be find in the Abbreviations/Nomenclature section at the end of the manuscript.

## Abbreviations

ACC	Acceptance
aERs	Adaptive Emotion Regulation Strategies
AERS	Adolescents’ Emotion Regulation Skills
BE	Binge Eating
B-EDI-3	Bulimia-EDI-3
BD-EDI-3	Body Dissatisfaction-EDI-3
BES	Binge Eating Scale
BES-TS	Binge Eating Scale-Total Score
BMI	Body Mass Index
CEM	Conformity Eating Motive
CERQ	Cognitive Emotion Regulation Questionnaire
ChEDEQ	Eating Disorder Examination Questionnaire for Children
CPS	Cognitive Problem Solving
DDE	Difficulty in Describing Emotions
DEa	Disordered Eating
DEBQ	Dutch Eating Behavior Questionnaire
DEBQK	Children’s Dutch Eating Behavior Questionnaire
DER	Difficulties in Emotional Regulation
DERS	Difficulties in Emotion Regulation Scale
DERS-SF	Difficulties in Emotion Regulation Scale-Short Form
DERS-TS	Difficulties in Emotion Regulation Scale-Total Score
DIS	Distraction
DT-EDI-3	Drive for Thinness-EDI-3
EA	Emotional Awareness
EaDS	Eating Disorder Symptoms
EaDR	Eating Disorder Risk
EAT-26	26-item Eating Attitude Test
EAT-26-TS	26-item Eating Attitude Test- Total Score
EB	Eating Behaviors
ECEM	Endorsement of Coping Eating Motives
ED	Emotion Dysregulation
EDI-3	Eating Disorder Inventory-3
EDI-3-TS	Eating Disorder Inventory-3- Total Score
EE	Emotional Expression
EI	Emotional Intelligence
EM	Eating Motives
EmE	Emotional Eating
EO	Exogenous Obesity
ER	Emotion Regulation
E-RDP	Energy-Rich Dietary Pattern
ERICA	Emotional Regulation Index for Children and Adolescents
ERQ	Emotion Regulation Questionnaire
ERQ-CS	Cognitive Reappraisal
ERQ-ES	Expressive Suppression
EU	Emotional Utilization
ExE	External Eating
F&V	Fruits and Vegetables
FEEL-KJ	Questionnaire to Assess Children’s and Adolescents’ Emotion Regulation Strategies
FEEL-KJ-TS	Questionnaire to Assess Children’s and Adolescents’ Emotion Regulation Strategies-Total Score
FFQ	Food Frequency Questionnaire
HC	Healthy Controls
HF	Hedonic Foods
K-PEMS	19-item Kids Palatable Eating Motive Scale
LC	Lean Controls
LEA	Lack of Emotional Awareness
LOC	Loss of Control
mERs	Maladaptive Emotion Regulation Strategies
NCT	Need to Control Thoughts
N-DDP	Nutrient-Dense Dietary Pattern
NEG	Neglect
NMR	The Generalized Expectancy for Negative Mood Regulation Scale
NW	Normal weight
OA	Adolescents with obesity
OWA	Adolescents with overweight
PIGH	Put into Good Humor
P-OA	Problem-Oriented Action
P-REA	Positive Reappraisal
P-REF	Positive Refocusing
REQ	Regulation of Emotion Questionnaire
RNEEB	Regulating Negative Emotional Experience in a Behavioral Manner
RUM	Rumination
S-B	Self-Blame
S-DEV	Self-Devaluation
S-EBPA	Self-Efficacy for Being Physically Active
S-EHFC	Self-Efficacy for Healthy Food Choices
TAS-20	20-item Toronto Alexithymia Scale
TEIQue-ASF	Trait Emotional Intelligence Questionnaire—Adolescent Short Form
UW	underweight
YAQ	Short version of the Youth/Adolescent Questionnaire
YRBS	Youth Risk Behavior Survey
WIT	Withdrawal
WLEIS	Wong and Law Emotional Intelligence Scale
BF	Body Fat
CEM	Coping Eating Motives
CoEM	Conform Eating Motives
DERS	Difficulties in Emotion Regulation Scale
DR	Dietary Restraint
EAT-26	26-item Eating Attitude Test
EEM	Enhance Eating Motives
EI	Emotion Inhibition
EM	Eating Motives
EmE	Emotional Eating
ER	Emotion Regulation
ERea	Emotion Reactivity
ExE	External Eating
HW	Healthy Weight
LEA	Lack of Emotional Awareness
LOCef	Loss of Control in Eating Frequency
mERs	Maladaptive Emotion Regulation Strategies
OB	Obesity
OW	Overweighed
